# Lateralization of Brain Activation in Fluent and Non-Fluent Preschool Children: A Magnetoencephalographic Study of Picture-Naming

**DOI:** 10.3389/fnhum.2014.00354

**Published:** 2014-05-28

**Authors:** Paul F. Sowman, Stephen Crain, Elisabeth Harrison, Blake W. Johnson

**Affiliations:** ^1^Department of Cognitive Science, ARC Centre of Excellence for Cognition and its Disorders, Macquarie University, Sydney, NSW, Australia; ^2^Perception and Action Research Centre (PARC), Faculty of Human Sciences, Macquarie University, Sydney, NSW, Australia; ^3^Department of Linguistics, ARC Centre of Excellence for Cognition and its Disorders, Macquarie University, Sydney, NSW, Australia

**Keywords:** stuttering, magnetoencephalography, lateralization, source analysis, speech, preschool children, speech production, vocalization

## Abstract

The neural causes of stuttering remain unknown. One explanation comes from neuroimaging studies that have reported abnormal lateralization of activation in the brains of people who stutter. However, these findings are generally based on data from adults with a long history of stuttering, raising the possibility that the observed lateralization anomalies are compensatory rather than causal. The current study investigated lateralization of brain activity in language-related regions of interest in young children soon after the onset of stuttering. We tested 24 preschool-aged children, half of whom had a positive diagnosis of stuttering. All children participated in a picture-naming experiment whilst their brain activity was recorded by magnetoencephalography. Source analysis performed during an epoch prior to speech onset was used to assess lateralized activation in three regions of interest. Activation was significantly lateralized to the left hemisphere in both groups and not different between groups. This study shows for the first time that significant speech preparatory brain activation can be identified in young children during picture-naming and supports the contention that, in stutterers, aberrant lateralization of brain function may be the result of neuroplastic adaptation that occurs as the condition becomes chronic.

## Introduction

Stuttering is a disorder of speech fluency that presents itself between the ages of 2 and 4 years. In the preschool population, the incidence is approximately 5% and the prevalence in the general population is 1%.

An early and influential theory of the brain basis of stuttering holds that its underlying cause is anomalous hemispheric lateralization of the speech control centers. Specifically, Orton (1927) contends that, in contrast to fluent speakers, people who stutter (PWS) have bilateral representations for speech processes. In their schema, rather than a single dominant (left) hemisphere producing speech, in the person who stutters both hemispheres issue commands which, when not perfectly synchronized, cause the blocking and repetition of speech segments that characterize stuttering. Despite the substantial face validity of this hypothesis, extensive behavioral testing of motor behavior in stutterers provides scant support. Work in the 80s and 90s by Webster (1985, 1986) converged on the position that “*people who stutter have normal left hemisphere lateralization of the neural mechanisms for the control of speech and other forms of sequential movement*” (Webster, 1997), findings that concur with sodium amytal tests of cerebral dominance for speech and language in PWS. As described by Andrews et al. (1972) and Luessenhop et al. (1973), PWS respond to right- and left-sided carotid artery injections of sodium amytal in the same way as fluent speakers. Such direct evidence supports the contention that PWS have a normal pattern of hemispheric specialization for speech.

Despite such findings, the theory that abnormal speech control lateralization drives stuttering still has currency in the general discourse around stuttering (e.g., Kushner, 2012). The perpetuation of this idea is supported in part by the findings from brain imaging evidence that has emerged in the past 20 years (for review, see Brown et al., 2005). A common finding in a number of these studies is a shift of speech-related brain activity to the right hemisphere in adults who stutter. In their seminal PET study, Fox et al. (1996) report increased activation in the right hemisphere in a language task in developmental stutterers. This finding was subsequently replicated by Braun et al. (1997), who were able to differentiate between patterns of stuttered and fluent speech in the stutterers that they tested. Importantly, their results challenge the idea that abnormal laterality caused stuttering. By demonstrating that the left hemisphere was more active during the production of stuttered speech and the right more active with fluent speech, the authors were able to conclude that the primary dysfunction in stuttering is located in the left hemisphere. They further suggested that hyperactivation of the right hemisphere is therefore not the cause of stuttering, but rather a reflection of neuroplastic adaptation.

Such compensatory plasticity has a well-established precedent in the lesion literature where transference of function between hemispheres has been observed in (e.g., Weiller et al., 1995). Following on from the early PET studies of stuttering, a subsequent fMRI investigation by Preibisch et al. (2003) showed that overactivity in the right frontal operculum in PWS was negatively correlated with stuttering. Furthermore, this overactivation was evident even when speech tasks were not required. Taken together, these observations support the idea that overactivation in the right hemisphere seen with functional neuroimaging in PWS reflects a compensatory mechanism rather than being a manifestation of abnormal cerebral dominance for speech control (e.g., Braun et al., 1997; Preibisch et al., 2003; Chang et al., 2008; Lu et al., 2010).

There is a missing piece in this puzzle that might help adjudicate between causal and reactive origins for hemispheric activation anomalies in stuttering. Given that stuttering emerges most commonly in the preschool years, observation of normal hemispheric laterality of brain activity during speech production would support the thesis that increases in the right hemispheric activation in adults who stutter are the result of compensatory mechanisms developed over a lifetime of stuttering. At present, there is no functional brain imaging evidence from children near the age of onset of stuttering. The present study was designed to provide such evidence.

## Materials and Methods

### Subjects

This study was conducted with the approval of the Macquarie University Human Ethics Committee #HE29MAY2009-R06572. Preschool children who stutter (CWS) were recruited by newspaper advertisement. All were examined by a highly experienced speech pathologist (Elisabeth Harrison) who has more than 20 years of experience in the diagnosis and treatment of stuttering, prior to their inclusion in the study. Twelve children who were positively diagnosed as stutterers (CWS) were included in the study. The stutterers as a group were typical of the wider population of preschool age CWS in terms of the severity of their stuttering, i.e., all were in the range of mild–moderately severe with severity ratings between 3 and 6 (1 = no stuttering, 2 = extremely mild stuttering, 10 = extremely severe stuttering). This was expected since the distribution of stuttering severity is positively skewed in both children and adults (Bloodstein and Ratner, 2008, p. 2). Age- and sex-matched typically developing (TD) control subjects were recruited. The group of CWS consisted of 2 females and 10 males, mean age 50.8 months (range 35–64 months), the TD group consisted of 2 females and 10 males, mean age 51.7 months (range 27–66 months). All children were first language speakers of English and right handed.

### Task

Subjects performed a picture-naming task based on that presented in Levelt et al. (1998). Twenty colored picture stimuli were selected from the colorized Snodgrass and Vanderwart set (Rossion and Pourtois, 2004). Pictures (Table 1) were selected on the basis that their name consisted of a single syllable and the age of acquisition of their name was <3 years (Snodgrass and Yuditsky, 1996). A simple picture-naming task was chosen so that the findings of the current study could be compared with those previous seminal magnetoencephalography (MEG) studies of picture-naming in adults (Salmelin et al., 1994, 2000; Levelt et al., 1998) and also because simple, short, repeated vocalization tasks induce very few if any instances of stuttering even in chronic stutterers (Salmelin et al., 2000; Chang et al., 2009).

**Table 1:** **Pictures used in the naming task**.

Word	Age of acquisition (years)
Ear	2.13
Dog	2.23
Hand	2.24
Sun	2.34
House	2.41
Bed	2.42
Sock	2.44
Spoon	2.45
Cat	2.5
Door	2.55
Cup	2.68
Box	2.69
Shoe	2.72
Cake	2.73
Car	2.73
Book	2.79
Fish	2.84
Bird	2.87
Hat	2.9
Duck	2.93

Each subject received one training block to get acquainted with the procedure and to maximize name agreement across items. Subjects lay supine on a plinth in the magnetically shielded room and were presented with the picture-naming stimuli projected via a mirror onto a screen that was situated directly in the participant’s line of sight. The experimental presentation was controlled by the Presentation software package (Presentation 14.4, Neurobehavioral Systems, Albany, NY, USA).

Trials began with a white fixation cross appearing in the center of a black background. The duration of the fixation cross was randomly varied between 3000 and 4000 ms after which time, a picture appeared in the center of the screen. The subject was instructed to respond to the picture by naming it as quickly as possible. Vocal responses triggered a voice key connected to a directional microphone positioned on the ceiling of the magnetically shielded room above the subject’s head. Timestamps thus collected were used to determine vocal onset reaction times. Trials were terminated as soon as the voice key was triggered. The active response period was limited to 3000 ms. Stimuli were presented in blocks of 20 trials. A single block contained all of the 20 stimuli randomly shuffled prior to the start of the block. Subjects participated in one or two recording sessions.

### Magnetoencephalography

Brain magnetic fields were measured during picture-naming using a custom built pediatric 64-channel whole-head gradiometer MEG system. A detailed description of specifications of this device is available in Johnson et al. (2010).

Before subjects entered the magnetically shielded room for MEG data acquisition, their head shapes were recorded using a digitizing pen (Polhemus Fastrack, Colchester, VT, USA); approximately 200 randomly selected points were recorded for each subject’s head surface. The 3D locations of the five head position indicator (HPI) coils attached to a tightly fitting elastic cap, and the locations of three cardinal landmarks (the nasion and bilateral preauricular points) were also digitized. Each subject’s head position in the MEG dewar was measured at the start and end of each recording block from the five HPI coils.

Continuous data were acquired at a sampling rate of 1000 Hz and filtered online between 0.03 and 250 Hz. Fieldtrip (Oostenveld et al., 2011) and SPM8 (Litvak et al., 2011) were used for all offline data analyses. Offline, data were filtered (bandpass 1–40 Hz), epoched around the time of stimulus onset (−1000 to 1000 ms), and baseline corrected. Trials containing large amplitude artifacts were removed using the Fieldtrip visual artifact rejection method. Data for each recording block were co-registered with the individual headshape data and then transformed into a common sensor space (the average sensor space across blocks within subjects) using the method described by Knosche (2002) and implemented in Fieldtrip.

### Sensor space analysis

In order to test whether stuttering status affected the evoked response to picture-naming stimuli, we used topological inference to search the entire sensor space for differences between groups. Based on the random field theory, topological inference for MEG data has been implemented in SPM8 (Litvak et al., 2011) to correct for multiple statistical comparisons across *N*-dimensional spaces. Briefly, a 2D topographical representation of the evoked field for each sample of the time dimension across the epoch of interest is created. Here, we created a 64 × 64 pixel image for each of the samples between −1000 and 1000 ms around the stimulus onset. This allowed us to compare differences in both space and time, while correcting for the family wise error (FWE) rate across the multiple comparisons. These images were then taken to the second level of the classical SPM analysis and compared using a two-sample *t*-test. Significance threshold was set at *p* < 0.05 (FWE-corrected) to determine whether statistically significant differences between groups (CWS vs. TD) existed in the evoked response at the sensor level.

### Source analysis

Source analysis was performed in Matlab (2013b; MathWorks, Inc., Natick, MA, USA) using the SPM8 toolbox for M/EEG. A canonical cortical mesh derived from the MNI template was co-registered and warped, in a non-linear manner, to match the participant’s digitized headshape. Leadfields were computed using a single sphere volume conductor model. Source localization was then performed using a group inversion with multiple sparse priors (Friston et al., 2008b; Litvak and Friston, 2008) and the greedy search method (Friston et al., 2008a). This procedure results in a spatial projection of sensor data into (3D) brain space and considers brain activity as comprising a very large number of dipolar sources spread over the cortical sheet, with fixed locations and orientations (Litvak et al., 2011).

In order to minimize the potential for movement and EMG artifacts distorting the source estimation, trials were discarded in which the subject’s vocal reaction time was shorter than 700 ms. Based on the approach using MEG to measure language laterality developed by Tanaka et al. (2013), evoked activity for each dipolar source was estimated within a 300 ms Gaussian time window centered on 450 ms after onset of the picture. Given the latency difference for linguistic processing known to exist for young children compared to adults (e.g., Holcomb et al., 1992; Kraus et al., 1993) we chose to shift the window of interest suggested by Tanaka et al. (2013), 50 ms later. According to Levelt et al. (1998), brain activity related to speech planning begins 300 ms after the onset of a picture-naming stimulus.

3D volumetric source maps were smoothed with a full width at half maximum (FWHM) smoothing kernel and passed to a second level SPM analysis. A paired *t*-test comparing stimulus-locked induced source activation to baseline was performed across the whole sample in order to identify a common network for speech preparation. A two-sample *t*-test was also conducted between CWS and TD in order to identify any differences in activation between the groups. Resulting SPMs were corrected for FWE. The data were thresholded at the critical FWE *t*-value and statistically significant difference clusters projected onto a template brain for visualization using xjView^1^

### ROI analysis

In order to test whether there was any effect of group or hemisphere on any of the activations within the ROIs, we performed a multivariate, repeated measures ANOVA on the between subjects factor Group (CWS or TD) and the within subjects factor Hemisphere (left or right) across the three ROIs, which were included as separate variates. This analysis was performed using IBM^®^ SPSS^®^ Statistics version 21.

### Laterality

In order to assess the degree of lateralization of the speech preparatory process, ROI masks for both hemispheres were constructed using the AAL atlas (Tzourio-Mazoyer et al., 2002) via the wfu_pickatlas toolbox (Maldjian et al., 2003). Following the procedure of Tanaka et al. (2013), we used anatomically defined ROIs that consisted of the supramarginal gyrus (SMG), superior temporal gyrus (STG), and the inferior frontal gyrus (IFG). We chose these areas in line with the core language network presented in Tanaka et al. (2013), that were based on previous language lateralization MEG studies (Bowyer et al., 2005; McDonald et al., 2009). Furthermore, these areas are the key areas in which previous functional imaging studies (e.g., De Nil et al., 2008) have shown there to be laterality anomalies in stuttering subjects. Tanaka et al. (2013) examined the opercular and triangular parts of the IFG separately whereas we chose to create a single ROI that consisted of the triangular, opercular, and orbital parts of the IFG in a single ROI that could be considered to represent Broca’s area and its right hemisphere homolog. Volumetric functional images were masked using these ROIs and then thresholded at the 25% maximal amplitude across all ROIs. Using the REX toolbox^2^, the mean voxel amplitude within these masks was extracted for all subjects. The laterality index (LI) was then calculated using the formula “left − right/left + right.” Therefore, LI varies continuously from −1 for pure right hemisphere dominance to +1 for pure left hemisphere dominance.

## Results

### Number of trials

The average total number of trials contributing to the analysis was 190 for the PWS and 160 for the TD. There was no significant difference between the two groups in terms of trial numbers (*p* = 0.16). The mean reaction time (mean ± SEM) for CWS was 1239 ± 64 and 1278 ± 72 ms for TD (*p* = 0.68).

### Sensor space analysis

**Figure 1:**
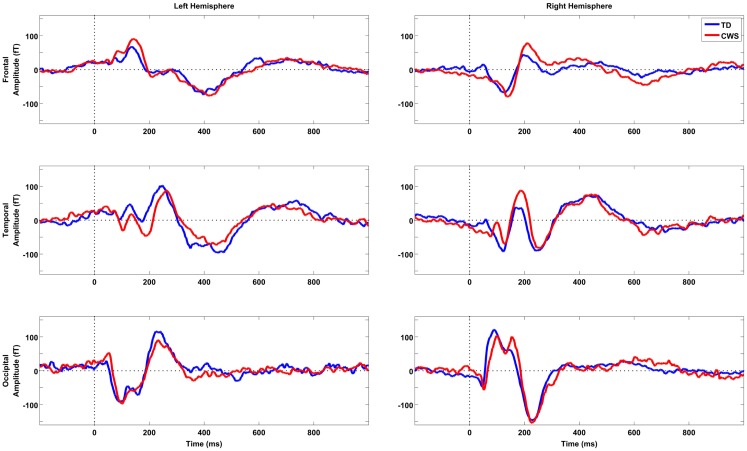
**Grand mean sensor space activations for six sample sensors taken from frontal, temporal, and occipital locations in the left and right hemispheres, respectively**. TD subjects are depicted in blue (*n* = 12) and CWS in red (*n* = 12). Vertical dotted lines represent picture-naming stimulus onset time.

Following the onset of the picture-naming stimulus, sensor level waveforms were characterized by an m100/200 complex, which was largest over occipital areas – consistent with early visual activation. A later component, peaking around 450 ms, was evident bilaterally in temporal areas and in the left frontal region. This pattern of activation is illustrated in the grand mean sensor plots in Figure 1. Sensor space SPMs found no significant between group differences (CWS vs. TD).

### Source space analysis

**Figure 2:**
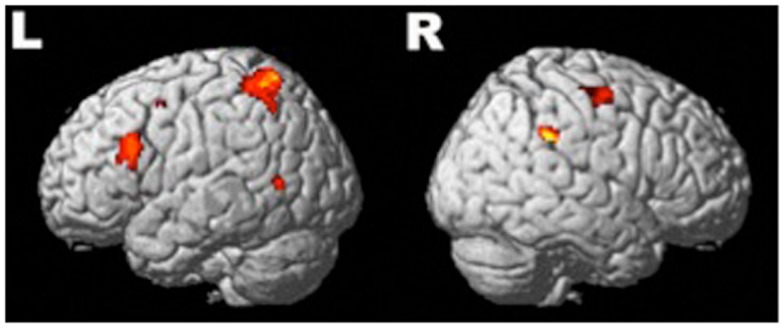
**Significant (active > baseline) regions of activation during speech preparation across all subjects (*n* = 24)**. Four clusters of activation survived correction (FWE) in the left hemisphere and two in the right. Description of the locations is available in Table 2.

Compared to baseline, there were six significant activation clusters in the brain during the epoch 300–600 ms after the onset of the naming stimulus (Figure 2). Four of these clusters were in the left hemisphere. In total, there were 1049 significantly activated voxels in the left hemisphere and 130 in the right (Table 2). In the left hemisphere, the largest cluster was in primary somatosensory and somatosensory association areas. It encompassed part of the posterior frontal lobe intersecting with Brodmann areas 3 and 2 and extended into the anterior-superior and inferior parietal lobe, intersecting with Brodmann areas 7, 5, and 40. The second largest cluster in the left hemisphere was centered on the triangular part of the IFG. This cluster overlaps with the representation of Broca’s area (Brodmann areas 45, 46, and 9). Two other small clusters were significantly active, one in the middle temporal gyrus intersecting with Brodmann area 39 and another in the supplementary motor area [SMA (Brodmann area 6)].

**Table 2:** **MNI coordinates and anatomical labels of FWE-corrected brain sources thresholded at *T* > 5.2 as revealed by task-baseline contrast**.

Cluster size (voxels)	Lobe	Area	Hemisphere	Brodmann areas	Peak intensity	MNI coordinates at peak (mm)
718	Parietal	Precuneus, superior parietal lobule, inferior parietal lobule, paracentral lobule	L	7, 5, 40, 3, 2	6.6	−18 −52 58
		Postcentral gyrus	
214	Frontal	Middle frontal gyrus, inferior frontal gyrus	L	9, 46, 45	5.6	−44 22 30
107	Parietal	Inferior parietal lobule, supramarginal gyrus	R	40	6.0	54 −42 36
100	Frontal	Precentral gyrus, middle frontal gyrus	R	6	5.8	34 −10 54
30	Temporal	Middle temporal gyrus	L	39	5.7	−52 −62 6
10	Frontal	Middle frontal gyrus	L	6	5.4	−32 4 50

In the right hemisphere, there were two significant clusters: the largest was in the SMG intersecting with Brodmann area 40. The other significant cluster was within the SMA Brodmann area 6. There were no significant activation differences between groups.

### ROI analysis

There was a significant main effect of Hemisphere for all ROIs [IFG: *F*_(1,22)_ = 32, *p* < 0.001; SMG: *F*_(1,22)_ = 35, *p* < 0.001; STG: *F*_(1,22)_ = 36, *p* < 0.001] with the level of activation being significantly greater in the left vs. the right hemisphere. There was no significant effect of Group or interaction between Group and Hemisphere.

### Lateralization

For all subjects, activity was lateralized to the left for all ROIs (Table 3). Lateralization was significantly to the left for all ROIs and there was no significant difference between groups (Table 4).

**Table 3:** **Laterality indices (LI) for all subjects (CWS and TD) across three ROIs**.

Subject	IFG		STG		SMG	
	CWS	TD	CWS	TD	CWS	TD
1	0.16	0.19	0.36	0.23	0.18	0.14
2	0.17	0.20	0.15	0.18	0.01	0.11
3	0.16	0.18	0.14	0.25	0.07	0.22
4	0.19	0.18	0.27	0.38	0.26	0.22
5	0.17	0.20	0.23	0.28	0.22	0.19
6	0.17	0.18	0.55	0.27	0.55	0.28
7	0.17	0.18	0.14	0.30	0.08	0.30
8	0.15	0.19	0.28	0.21	0.11	0.26
9	0.17	0.17	0.20	0.22	0.19	0.13
10	1.00	1.00	0.31	0.60	0.36	0.50
11	0.18	0.20	0.29	0.23	0.09	0.12
12	1.00	0.15	0.35	0.34	0.33	0.29

*IFG, inferior frontal gyrus; STG, superior temporal gyrus; SMG, supramarginal gyrus*.

**Table 4:** **Mean (±SEM) laterality indices (LI) for CWS and TD across three ROIs**.

ROI	CWS	TD	Two-sample *t*-test	All subjects	One-sample *t*-test
IFG	0.31 ± 0.09	0.25 ± 0.07	*p* = 0.63	0.28 ± 0.06	*p* < 0.001
STG	0.27 ± 0.03	0.29 ±0.03	*p* = 0.68	0.28 ± 0.02	*p* < 0.001
SMG	0.21 ± 0.04	0.23 ± 0.03	*p* = 0.64	0.22 ± 0.03	*p* < 0.001

*IFG, inferior frontal gyrus; STG, superior temporal gyrus; SMG, supramarginal gyrus. Two-sample *t*-test values refer to tests between controls and stutterers. One-sample *t*-tests refer to tests of the whole sample’s LI against 0 (no laterality). LI varies continuously from −1 for pure right hemisphere dominance to +1 for pure left hemisphere dominance*.

## Discussion

The current results are the first functional brain imaging data of overt speech production in preschool-aged CWS. This is an important contribution to a literature based on results from older children and adults, whose brain functions have had many years to develop compensatory strategies.

There is a long history of attributing the cause of stuttering to atypical laterality of speech/language function. The roots of this theory can be traced back to publications in the early twentieth century by Orton (1927) and Travis (1931), hence the lateralization theory of stuttering often being referred to as the Orton–Travis theory. They posited that a failure in development of normal cerebral dominance would lead to cascade of events: competition between the hemispheres, an incoordination of outputs and interruption of fluent speech. Even though the early attempts to test this theory experimentally were inconclusive (Bryngelson, 1935, 1939; Heltman, 1940) and a number of negative findings followed, e.g., Dorman and Porter (1975), interest in the theory has persisted, most likely because of its parsimonious appeal and the persistence of anecdotal evidence suggesting that forced changes in handedness for writing – a common educational practice in the early twentieth century (Kushner, 2012) – gave rise to stuttering or that left handedness conveys a higher risk for stuttering. Indeed, it is still common to find examples such claims as “Most stammering children are left-handed” (du Plessix Gray, 2012) or “In fact, just as human speech – stuttering in particular – is related to cerebral laterality (most stutterers are left-handed)…” (Shell, 2006), in the popular press, despite significant evidence to the contrary (Records et al., 1977; Webster and Poulos, 1987; Ardila et al., 1994; Salihovic and Sinanovic, 2000). While the burgeoning neuroimaging literature on stuttering has conferred some support for the idea that anomalous laterality is the cause of stuttering, such imaging data cannot provide a causal link. Indeed, it has long been contended that right hemispheric overactivations represent reactions to, or compensations for stuttering, rather than being causative agents. For this reason, anatomical or functional demonstrations of normal laterality in young stutterers are powerful evidence against a laterality origin for stuttering and would support a reactive origin for the changes in both functional and structural laterality changes seen in adult stutterers. Our data strongly support the contention that the laterality anomalies of older stutterers reflect compensatory shifting of function rather than an underlying causal dysfunction. This conclusion is supported by a recent neuroanatomical study that reported no differences in right–left asymmetries between 9- and 12-year-old stuttering boys and a matched cohort of controls (Chang et al., 2008).

An important caveat to this conclusion comes from the fact that a significant proportion of those children who begin to stutter will spontaneously recover [up to 80% by some estimations (Yairi and Ambrose, 1992, 1999; Yairi et al., 1993; Kalinowski et al., 2002)]. With this in mind, it is possible that our child participants may be quite different neurologically from children whose stuttering persists into adulthood. That is, while the sample we tested were all current stutterers at the time of our investigation, it must be expected that most of them would not continue to be stutterers into adulthood and therefore a large proportion of our sample consists of stutterers who will recover precisely because they do not have the underlying abnormal laterality that leads to persistent developmental stuttering. This possibility is encapsulated well in the work of Shell (2006) who states, “Particularized lateralization among human children develops ontogenetically at around the time they are learning to speak. Some researchers think this fact may explain why so many children (3–4%) “stumble” in speech and then “outgrow” the problem when lateralization is fully developed. Those children who do not fully lateralize are, according to this view, the children who are the “real” stutterers (about 1%).” Arguing against this possibility, the study by Chang et al. (2008) showed that the brains of recovered stutterers were more like those of current stutterers than control subjects, an observation that suggests that the occurrence of recovery likely reflects initial severity rather than reflecting the existence of a neurologically distinct subgroup. For this reason, we believe our data are still likely to reflect the true status of laterality in the early stages of stuttering, at least in regard to speech production.

The possibility remains though that anomalous laterality of other speech or language-related brain functions might exist in the early stages of stuttering. Indeed, a recent study using near infrared spectroscopy suggests that this may be the case in regard to some aspects of auditory language processing (Sato et al., 2011). Future studies looking to characterize brain activation anomalies in young CWS should include longitudinal following of the subjects so that retrospective analysis of those subjects whose stuttering does not resolve might be carried out. Given a large enough initial cohort, this approach would allow researchers to control for possible heterogeneities within the cohort.

While the interpretation of child MEG data in source space must be considered in light of the inherent uncertainties that govern solutions to the inverse problem, the concordance between the results of the whole-brain analysis presented herein and previous MEG studies of speech in adult subjects suggests that these findings are robust. In the time after early visual processing, and consistent with articulatory planning for speech (Levelt et al., 1998), a strongly left-lateralized brain network was activated. This network consisted of inferior frontal, parietal, and temporal nodes largely consistent with previous MEG studies that have examined speech or speech planning (e.g., Carota et al., 2010). Notably, our results show a distinctly left-lateralized inferior frontal activation, and premotor activity in areas consistent with the SMA activity seen in previous studies (Salmelin et al., 1994, 2000). Furthermore, like the study of Salmelin et al. (2000), the activity we saw in the SMA was right lateralized. We also observed significant parietal lobe activations in our study which, while not consistent with the fMRI literature on overt speech (Indefrey and Levelt, 2004), is consistent with similar MEG studies, which have consistently found activation in both inferior and superior parietal lobes (Salmelin et al., 1994; Levelt et al., 1998; Hulten et al., 2009) including Brodmann area 7 (Carota et al., 2010), which was the most active parietal locus in our findings. Carota et al. (2010) suggest that activation in Brodmann area 7 during speech planning is indicative of the parietal cortex’s key role in monitoring motor intention in language.

Our conclusions regarding the lack of laterality differences must be considered within the scope of the limited part of the speech planning process that we have examined. It is important to emphasize that the time window beyond 600 ms was not taken into inversion analysis and, given that the average vocal reaction time was longer than 1000 ms, there remains a significant epoch in which laterality differences might manifest. It is, however, important to note that articulatory mouth movement begins significantly earlier than the onset of overt speech – similar studies to the current one suggest this difference is in the order of 300 ms in adult subjects (Salmelin et al., 2000) hence the speech planning time is not as long as the reaction time as measured by voice key as in the current study. Future development of devices, which allow real-time monitoring of articulatory movements within the MEG environment (e.g., Lau, 2013) should allow for articulatory artifacts to be controlled much more precisely and remove a number of the limitations surrounding the time frame in which brain processing of speech production might be measured.

A number of previous neuroimaging studies using hemodynamic techniques (PET, fMRI) have shown there to be differences between stutterers and non-stutterers in the activation strength of various cortical and subcortical sources (e.g., Fox et al., 1996, 2000; Braun et al., 1997; De Nil et al., 2000, 2008; Ingham et al., 2000; Neumann et al., 2003; Preibisch et al., 2003; Giraud et al., 2008; Watkins et al., 2008; Chang et al., 2009; Kell et al., 2009; Sakai et al., 2009; Loucks et al., 2011); (for review, see De Nil and Kroll, 2001; Ingham, 2001; Fox, 2003; Brown et al., 2005). However, our study did not find any difference between source activation strength between CWS and TD. This difference may reflect a difference between neuromagnetic approaches to source imaging compared to hemodynamic imaging. Indeed, most previous MEG studies of stuttering have not attempted to analyze differences in source activations, rather utilizing the inherent temporal advantage of MEG to illustrate differences in auditory evoked activations (Salmelin et al., 1998; Beal et al., 2010; Kikuchi et al., 2011b) and temporal dynamics (Salmelin et al., 2000; Biermann-Ruben et al., 2005) or location of discrete dipole sources (Salmelin et al., 2000). Only a single previous study has demonstrated the ability of MEG to characterize cortical activation patterns in stuttering using a distributed sources model, and that was in a single adult subject (Sowman et al., 2012). Our primary aim was to use the other advantages of MEG (passivity, lack of noise, and reduced need for enclosure of the participant) to investigate laterality in children. A previous MEG study using a similar approach has shown that left dominance of parietotemporal coherence in theta band activity is specifically correlated with higher performance of language-related tasks in preschool children (Kikuchi et al., 2011a). The current study also demonstrates the utility and possible sensitivity of MEG-based measures for characterizing laterality in developmental language disorders.

In conclusion, we have demonstrated that in the very early stages of stuttering development, the preparation for speech is not characterized by anomalous lateralization of brain activations. This evidence gives weight to the hypothesis that the right hemispheric biases in chronic stuttering are due to neuroplastic adaptations rather than being an underlying primary source of dysfunction.

## Conflict of Interest Statement

The authors declare that the research was conducted in the absence of any commercial or financial relationships that could be construed as a potential conflict of interest.

## Footnotes

^1^http://www.alivelearn.net/xjview.

^2^http://gablab.mit.edu/
